# Supplementary material for: Combined General/Epidural Anesthesia vs. General Anesthesia on Postoperative Cytokines: A Review and Meta-Analysis
Source: Cancers (Basel). 2025 May 15;17(10):1667. doi: 10.3390/cancers17101667 (PMC12109670; doi:10.3390/cancers17101667)
Supplement: Supplementary file 1 [file cancers-17-01667-s001.zip › cancers-3564613-supplementary.pdf]

Supplementary Table S1: summary of the meta-analysis outcomes

Outcome	**Number of studies**	**Standardized mean difference (95% CI)**	**I^2**	**Egger's regression**	**Trim and fill missing studies**
IL-6 @ 24 hours	16	-1.99 (-2.67, -1.31)	97%	<0.01	5
Cancer surgery	12	-1.96 (-2.60, -1.21)	93%	<0.01	3
TNF @ 24 hours	9	-1.13 (-1.74, -0.51)	92%	0.11	1
Cancer surgery	7	-0.89 (-1.47, -0.32)	89%	0.05	0
CRP @ 24 hours	7	-0.61 (-0.97, -0.24)	71%	0.11	0
Cancer surgery	6	-0.72 (-1.07, -0.36)	63%	0.06	0
IL-6 @ end of surgery	10	-1.28 (-2.35, -0.21)	95%	<0.01	0
Cancer surgery	7	-1.88 (-2.90, -0.87)	94%	0.01	0
TNF @ end of surgery	7	-0.80 (-1.53, -0.08)	93%	<0.01	0
Cancer surgery	5	-0.08 (-0.39, 0.23)	54%	-	-
CRP @ end of surgery	5	-1.60 (-2.78, -0.42)	96%	0.15	0
Cancer surgery	4	-1.96 (-3.37, -0.55)	96%	0.27	0
Cortisol @ 24 hours	8	-0.82 (-1.22, -0.43)	78%	0.86	0
Cortisol @ end of surgery	7	-1.94 (-3.19, -0.68)	96%	<0.01	0
IL1b	5	-1.70 (-2.66, -0.74)	93%	0.47	0
IL4	5	0.83 (-1.26, 2.91)	93%	-	-
IL8	5	-1.53 (-2.04, -1.03)	79%	0.84	0
IL10	7	1.06 (-0.52, 2.64)	79%	-	-

A

Study or Subgroup	TEA+GA Mean	TEA+GA SD	TEA+GA Total	GA Mean	GA SD	GA Total	Weight	Std. Mean Difference IV, Random, 95% CI	Year
**1.5.1 abdominal/pelvic**									
Moselli 2011	171.15	15.89	18	1,226.19	416.67	17	5.8%	−3.55 [−4.65, −2.45]	2011
Hadimioglu 2012	4,651.16	3,455.15	21	6,245.85	5,847.17	25	6.5%	−0.32 [−0.90, 0.26]	2012
Xu 2014	26.75	6.84	20	33.6	8.32	20	6.4%	−0.88 [−1.53, −0.23]	2014
Fares 2014	55.2	24.6	15	80.6	13.7	15	6.2%	−1.24 [−2.03, −0.45]	2014
Hou 2019	140.2	20.58	60	177.22	17.03	60	6.6%	−1.95 [−2.38, −1.51]	2019
Yan 2019	95.2	6.8	37	102.5	9.1	37	6.6%	−0.90 [−1.38, −0.42]	2019
Duque 2019	38.8	5	20	71.5	7	19	5.3%	−5.29 [−6.68, −3.90]	2019
Xu 2020	21.5	1.5	55	28	2.4	55	6.5%	−3.23 [−3.80, −2.65]	2020
Elsayed 2020	17	2.9	20	19.4	3.6	20	6.4%	−0.72 [−1.36, −0.08]	2020
**Subtotal (95% CI)**			**266**			**268**	**56.1%**	**−1.90 [−2.71, −1.10]**	
Heterogeneity: Tau² = 1.36; Chi² = 111.70, df = 8 (P < 0.00001); I² = 93%									
Test for overall effect: Z = 4.65 (P < 0.00001)									
**1.5.2 thoracic**									
Yokoyama 2005	350	196.77	15	372.58	222.58	15	6.3%	−0.10 [−0.82, 0.61]	2005
Caputo 2009	60.55	7.09	36	77.69	7.91	38	6.5%	−2.25 [−2.84, −1.67]	2009
Gu 2015	0.1173	0.0255	14	0.14	0.0563	15	6.3%	−0.50 [−1.24, 0.24]	2015
Tan 2016	86.94	13.6	53	187.25	21.45	53	6.1%	−5.55 [−6.40, −4.69]	2016
Cong 2020	109.52	25.6	40	152.6	30.5	40	6.5%	−1.52 [−2.02, −1.02]	2020
**Subtotal (95% CI)**			**158**			**161**	**31.7%**	**−1.97 [−3.52, −0.42]**	
Heterogeneity: Tau² = 3.02; Chi² = 111.32, df = 4 (P < 0.00001); I² = 96%									
Test for overall effect: Z = 2.49 (P = 0.01)									
**1.5.3 upper GI/hepatic**									
Sun 2016	95.49	12.7	36	101.64	10.25	36	6.6%	−0.53 [−1.00, −0.06]	2016
Wang 2019	27.24	1.33	20	33.58	1.42	20	5.6%	−4.52 [−5.73, −3.30]	2019
**Subtotal (95% CI)**			**56**			**56**	**12.1%**	**−2.48 [−6.39, 1.43]**	
Heterogeneity: Tau² = 7.74; Chi² = 36.14, df = 1 (P < 0.00001); I² = 97%									
Test for overall effect: Z = 1.24 (P = 0.21)									
**Total (95% CI)**			**480**			**485**	**100.0%**	**−1.99 [−2.67, −1.31]**	
Heterogeneity: Tau² = 1.76; Chi² = 266.46, df = 15 (P < 0.00001); I² = 94%									
Test for overall effect: Z = 5.76 (P < 0.00001)									
Test for subgroup differences: Chi² = 0.08, df = 2 (P = 0.96), I² = 0%									

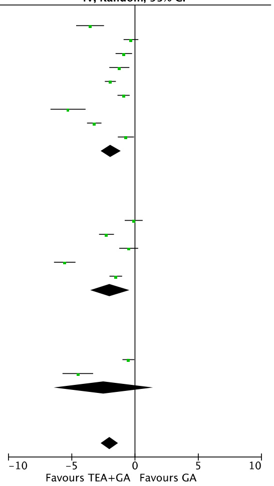

B

Study or Subgroup	TEA+GA Mean	TEA+GA SD	TEA+GA Total	GA Mean	GA SD	GA Total	Weight	Std. Mean Difference IV, Random, 95% CI	Year
**1.6.1 <180 minutes**									
Hadimioglu 2012	4,651.16	3,455.15	21	6,245.85	5,847.17	25	6.5%	−0.32 [−0.90, 0.26]	2012
Xu 2014	26.75	6.84	20	33.6	8.32	20	6.4%	−0.88 [−1.53, −0.23]	2014
Gu 2015	0.1173	0.0255	14	0.14	0.0563	15	6.3%	−0.50 [−1.24, 0.24]	2015
Sun 2016	95.49	12.7	36	101.64	10.25	36	6.6%	−0.53 [−1.00, −0.06]	2016
Hou 2019	140.2	20.58	60	177.22	17.03	60	6.6%	−1.95 [−2.38, −1.51]	2019
Yan 2019	95.2	6.8	37	102.5	9.1	37	6.6%	−0.90 [−1.38, −0.42]	2019
Wang 2019	27.24	1.33	20	33.58	1.42	20	5.6%	−4.52 [−5.73, −3.30]	2019
Xu 2020	21.5	1.5	55	28	2.4	55	6.5%	−3.23 [−3.80, −2.65]	2020
Elsayed 2020	17	2.9	20	19.4	3.6	20	6.4%	−0.72 [−1.36, −0.08]	2020
**Subtotal (95% CI)**			**283**			**288**	**57.3%**	**−1.44 [−2.17, −0.71]**	
Heterogeneity: Tau² = 1.14; Chi² = 111.92, df = 8 (P < 0.00001); I² = 93%									
Test for overall effect: Z = 3.86 (P = 0.0001)									
**1.6.2 > 180 minutes**									
Yokoyama 2005	350	196.77	15	372.58	222.58	15	6.3%	−0.10 [−0.82, 0.61]	2005
Caputo 2009	60.55	7.09	36	77.69	7.91	38	6.5%	−2.25 [−2.84, −1.67]	2009
Moselli 2011	171.15	15.89	18	1,226.19	416.67	17	5.8%	−3.55 [−4.65, −2.45]	2011
Fares 2014	55.2	24.6	15	80.6	13.7	15	6.2%	−1.24 [−2.03, −0.45]	2014
Tan 2016	86.94	13.6	53	187.25	21.45	53	6.1%	−5.55 [−6.40, −4.69]	2016
Duque 2019	38.8	5	20	71.5	7	19	5.3%	−5.29 [−6.68, −3.90]	2019
Cong 2020	109.52	25.6	40	152.6	30.5	40	6.5%	−1.52 [−2.02, −1.02]	2020
**Subtotal (95% CI)**			**197**			**197**	**42.7%**	**−2.73 [−4.07, −1.40]**	
Heterogeneity: Tau² = 3.04; Chi² = 129.72, df = 6 (P < 0.00001); I² = 95%									
Test for overall effect: Z = 4.01 (P < 0.0001)									
**Total (95% CI)**			**480**			**485**	**100.0%**	**−1.99 [−2.67, −1.31]**	
Heterogeneity: Tau² = 1.76; Chi² = 266.46, df = 15 (P < 0.00001); I² = 94%									
Test for overall effect: Z = 5.76 (P < 0.00001)									
Test for subgroup differences: Chi² = 2.76, df = 1 (P = 0.10), I² = 63.7%									

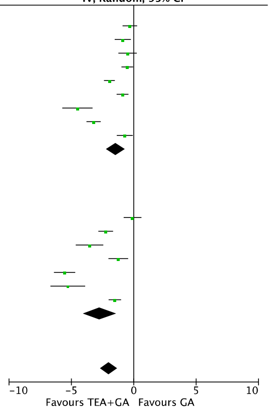

C

Study or Subgroup	TEA+GA Mean	TEA+GA SD	TEA+GA Total	GA Mean	GA SD	GA Total	Weight	Std. Mean Difference IV, Random, 95% CI	Year
**1.3.1 cancer surgery**									
Yokoyama 2005	350	196.77	15	372.58	222.58	15	8.5%	−0.10 [−0.82, 0.61]	2005
Moselli 2011	171.15	15.89	18	1,226.19	416.67	17	7.5%	−3.55 [−4.65, −2.45]	2011
Xu 2014	26.75	6.84	20	33.6	8.32	20	8.6%	−0.88 [−1.53, −0.23]	2014
Fares 2014	55.2	24.6	15	80.6	13.7	15	8.3%	−1.24 [−2.03, −0.45]	2014
Gu 2015	0.1173	0.0255	14	0.14	0.0563	15	8.4%	−0.50 [−1.24, 0.24]	2015
Sun 2016	95.49	12.7	36	101.64	10.25	36	8.9%	−0.53 [−1.00, −0.06]	2016
Duque 2019	38.8	5	20	71.5	7	19	6.8%	−5.29 [−6.68, −3.90]	2019
Hou 2019	140.2	20.58	60	177.22	17.03	60	9.0%	−1.95 [−2.38, −1.51]	2019
Yan 2019	95.2	6.8	37	102.5	9.1	37	8.9%	−0.90 [−1.38, −0.42]	2019
Wang 2019	27.24	1.33	20	33.58	1.42	20	7.3%	−4.52 [−5.73, −3.30]	2019
Cong 2020	109.52	25.6	40	152.6	30.5	40	8.9%	−1.52 [−2.02, −1.02]	2020
Xu 2020	21.5	1.5	55	28	2.4	55	8.8%	−3.23 [−3.80, −2.65]	2020
**Subtotal (95% CI)**			**350**			**349**	**100.0%**	**−1.91 [−2.60, −1.21]**	
Heterogeneity: Tau² = 1.35; Chi² = 152.45, df = 11 (P < 0.00001); I² = 93%									
Test for overall effect: Z = 5.38 (P < 0.00001)									
**1.3.2 non-cancer surgery**									
Caputo 2009	60.55	7.09	36	77.69	7.91	38	25.2%	−2.25 [−2.84, −1.67]	2009
Hadimioglu 2012	4,651.16	3,455.15	21	6,245.85	5,847.17	25	25.2%	−0.32 [−0.90, 0.26]	2012
Tan 2016	86.94	13.6	53	187.25	21.45	53	24.6%	−5.55 [−6.40, −4.69]	2016
Elsayed 2020	17	2.9	20	19.4	3.6	20	25.1%	−0.72 [−1.36, −0.08]	2020
**Subtotal (95% CI)**			**130**			**136**	**100.0%**	**−2.19 [−4.19, −0.20]**	
Heterogeneity: Tau² = 4.03; Chi² = 112.22, df = 3 (P < 0.00001); I² = 97%									
Test for overall effect: Z = 2.15 (P = 0.03)									
Test for subgroup differences: Chi² = 0.07, df = 1 (P = 0.79), I² = 0%									

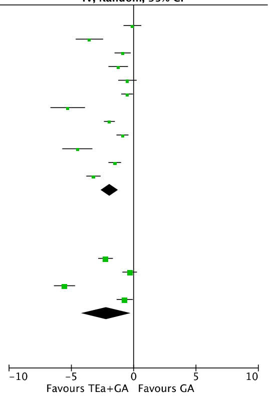

Supplementary Figure S1. Subgroup analysis of IL-6 at 24 hours after surgery. Forest plot of subgroup analysis based on (A) surgery type, (B) surgery length, and (C) cancer or non-cancer surgery.

Study or Subgroup	TEA+GA Mean	TEA+GA SD	TEA+GA Total	GA Mean	GA SD	GA Total	Weight	Std. Mean Difference IV, Random, 95% CI	Year
**2.3.1 cancer surgery**									
Yokoyama 2005	2.87	1.6	15	2.69	1.75	15	13.4%	0.10 [−0.61, 0.82]	2005
Moselli 2011	165.04	25.67	18	278.85	30.04	17	9.8%	−3.99 [−5.18, −2.80]	2011
Pi 2019	0.64	0.11	74	0.72	0.11	75	16.0%	−0.72 [−1.06, −0.39]	2019
Duque 2019	0.84	0.1	20	0.93	0.1	19	13.9%	−0.88 [−1.54, −0.22]	2019
Liu 2019	640.27	111.5	54	719.91	102.21	53	15.7%	−0.74 [−1.13, −0.35]	2019
Hou 2019	23.25	7.69	60	30.52	3.53	60	15.7%	−1.21 [−1.60, −0.82]	2019
Cong 2020	34.5	8.2	40	33.1	6.1	40	15.4%	0.19 [−0.25, 0.63]	2020
**Subtotal (95% CI)**			**281**			**279**	**100.0%**	**−0.89 [−1.47, −0.32]**	
Heterogeneity: $Tau^2 = 0.51$; $Chi^2 = 56.71$, df = 6 (P < 0.00001); $I^2 = 89\%$									
Test for overall effect: Z = 3.04 (P = 0.002)									
**2.3.2 non−cancer surgery**									
Hadimioglu 2012	283.46	496.08	21	3,637.8	944.88	25	49.2%	−4.26 [−5.34, −3.18]	2012
Tan 2016	43.58	5.18	53	43.95	3.09	53	50.8%	−0.09 [−0.47, 0.29]	2016
**Subtotal (95% CI)**			**74**			**78**	**100.0%**	**−2.14 [−6.23, 1.95]**	
Heterogeneity: $Tau^2 = 8.54$; $Chi^2 = 51.07$, df = 1 (P < 0.00001); $I^2 = 98\%$									
Test for overall effect: Z = 1.03 (P = 0.30)									

Test for subgroup differences: $Chi^2 = 0.35$, df = 1 (P = 0.55), $I^2 = 0\%$

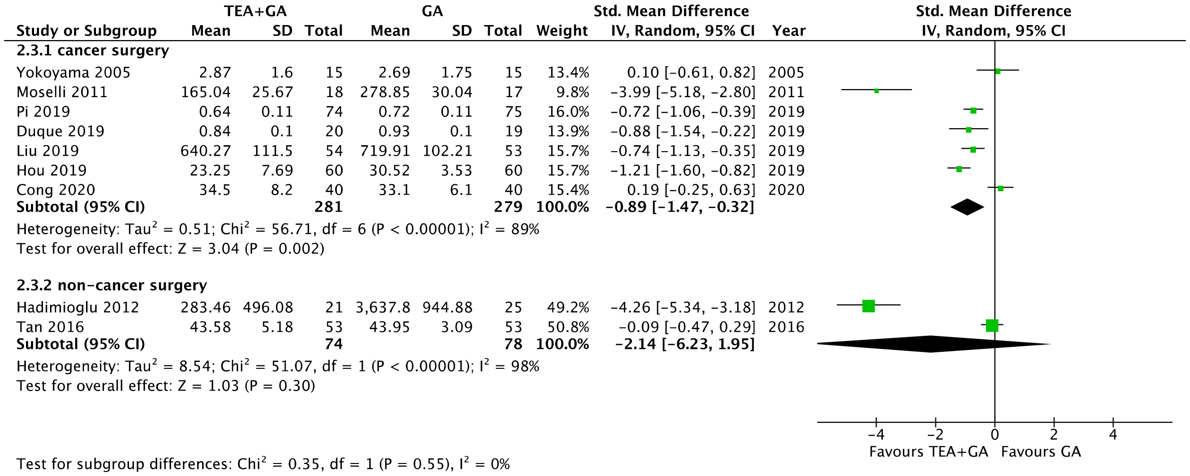

Supplementary Figure S2. Subgroup analysis of TNF- α at 24 hours after surgery.

Study or Subgroup	TEA+GA Mean	TEA+GA SD	TEA+GA Total	GA Mean	GA SD	GA Total	Weight	Std. Mean Difference IV, Random, 95% CI	Year
**3.3.1 cancer surgery**									
Yokoyama 2005	7.96	3.6	15	8.57	3.63	15	13.0%	−0.16 [−0.88, 0.55]	2005
Fant 2013	69	36	12	67	25	14	12.0%	0.06 [−0.71, 0.83]	2013
Pi 2019	1.94	0.16	74	2.12	0.15	75	22.0%	−1.16 [−1.50, −0.81]	2019
Duque 2019	12.4	0.9	20	13.4	1.1	19	14.0%	−0.98 [−1.65, −0.31]	2019
Liu 2019	1.95	0.144	54	2.097	0.154	53	20.6%	−0.98 [−1.38, −0.58]	2019
Yan 2019	94.4	7.4	34	99.6	9.7	36	18.5%	−0.59 [−1.07, −0.11]	2019
**Subtotal (95% CI)**			**209**			**212**	**100.0%**	**−0.72 [−1.07, −0.36]**	
Heterogeneity: $Tau^2 = 0.12$; $Chi^2 = 13.47$, df = 5 (P = 0.02); $I^2 = 63\%$									
Test for overall effect: Z = 3.98 (P < 0.0001)									
**3.3.2 non−cancer surgery**									
Sidiropoulou 2016	48.15	11.73	30	49.68	19.69	30	100.0%	−0.09 [−0.60, 0.41]	2016
**Subtotal (95% CI)**			**30**			**30**	**100.0%**	**−0.09 [−0.60, 0.41]**	
Heterogeneity: Not applicable									
Test for overall effect: Z = 0.36 (P = 0.72)									

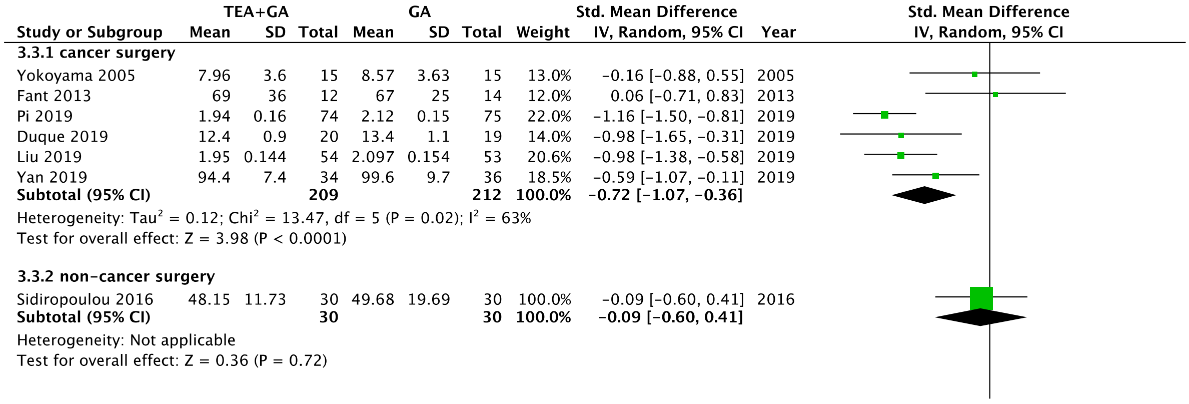

Sup[illegible]

A

Study or Subgroup	TEA+GA Mean	TEA+GA SD	TEA+GA Total	GA Mean	GA SD	GA Total	Weight	Std. Mean Difference IV, Random, 95% CI	Year
Yokoyama 2005	445.16	209.68	15	495.16	224.19	15	9.2%	-0.22 [-0.94, 0.49]	2005
Caputo 2009	24.74	3.07	36	27.1	3.42	38	9.5%	-0.72 [-1.19, -0.25]	2009
Hadimioglu 2012	1,860.47	3,322.26	21	7,308.97	5,980.07	25	9.3%	-1.08 [-1.71, -0.46]	2012
Fares 2014	123.9	14	15	218.3	10.5	15	6.6%	-7.42 [-9.56, -5.28]	2014
Gu 2015	0.094	0.0253	14	0.118	0.0378	15	9.2%	-0.72 [-1.48, 0.03]	2015
Sun 2016	114.75	11.88	36	123.77	11.07	36	9.5%	-0.78 [-1.26, -0.30]	2016
Tan 2016	67.47	7.32	53	47.59	8.36	53	9.4%	2.51 [2.00, 3.03]	2016
Duque 2019	83.5	4	20	96.1	8	19	9.1%	-1.97 [-2.75, -1.19]	2019
Cong 2020	94.1	21.1	40	118.5	32.5	40	9.5%	-0.88 [-1.34, -0.42]	2020
Xu 2020	24.8	1.6	55	31.9	2.6	55	9.4%	-3.27 [-3.84, -2.69]	2020
Okuda 2022	30	7.96	30	42.8	12.77	30	9.4%	-1.19 [-1.74, -0.64]	2022
**Total (95% CI)**			**335**			**341**	**100.0%**	**-1.26 [-2.22, -0.30]**	

Heterogeneity: $Tau^2 = 2.49$; $Chi^2 = 280.73$, df = 10 ($P < 0.00001$); $I^2 = 96\%$
Test for overall effect: Z = 2.56 (P = 0.01)

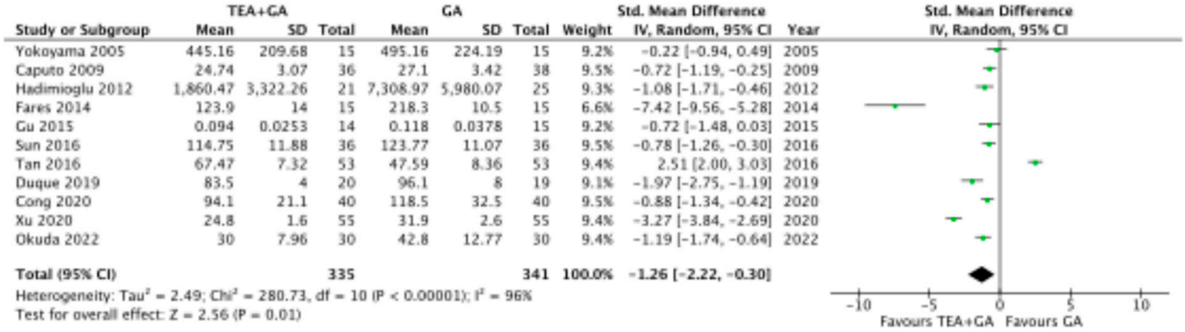

B

Study or Subgroup	TEA+GA Mean	TEA+GA SD	TEA+GA Total	GA Mean	GA SD	GA Total	Weight	Std. Mean Difference IV, Random, 95% CI	Year
**1.4.1 cancer surgery**									
Yokoyama 2005	445.16	209.68	15	495.16	224.19	15	12.9%	-0.22 [-0.94, 0.49]	2005
Fares 2014	123.9	14	15	218.3	10.5	15	7.5%	-7.42 [-9.56, -5.28]	2014
Gu 2015	0.094	0.0253	14	0.118	0.0378	15	12.8%	-0.72 [-1.48, 0.03]	2015
Sun 2016	114.75	11.88	36	123.77	11.07	36	13.6%	-0.78 [-1.26, -0.30]	2016
Duque 2019	83.5	4	20	96.1	8	19	12.7%	-1.97 [-2.75, -1.19]	2019
Xu 2020	24.8	1.6	55	31.9	2.6	55	13.4%	-3.27 [-3.84, -2.69]	2020
Cong 2020	94.1	21.1	40	118.5	32.5	40	13.7%	-0.88 [-1.34, -0.42]	2020
Okuda 2022	30	7.96	30	42.8	12.77	30	13.4%	-1.19 [-1.74, -0.64]	2022
**Subtotal (95% CI)**			**225**			**225**	**100.0%**	**-1.75 [-2.61, -0.90]**	
Heterogeneity: $Tau^2 = 1.35$; $Chi^2 = 97.62$, df = 7 ($P < 0.00001$); $I^2 = 93\%$									
Test for overall effect: Z = 4.01 (P < 0.0001)									
**1.4.2 non-cancer surgery**									
Caputo 2009	24.74	3.07	36	27.1	3.42	38	33.5%	-0.72 [-1.19, -0.25]	2009
Hadimioglu 2012	1,860.47	3,322.26	21	7,308.97	5,980.07	25	33.1%	-1.08 [-1.71, -0.46]	2012
Tan 2016	67.47	7.32	53	47.59	8.36	53	33.4%	2.51 [2.00, 3.03]	2016
**Subtotal (95% CI)**			**110**			**116**	**100.0%**	**0.24 [-2.02, 2.50]**	
Heterogeneity: $Tau^2 = 3.90$; $Chi^2 = 107.96$, df = 2 ($P < 0.00001$); $I^2 = 98\%$									
Test for overall effect: Z = 0.21 (P = 0.83)									

Test for subgroup differences: $Chi^2 = 2.62$, df = 1 (P = 0.11), $I^2 = 61.8\%$

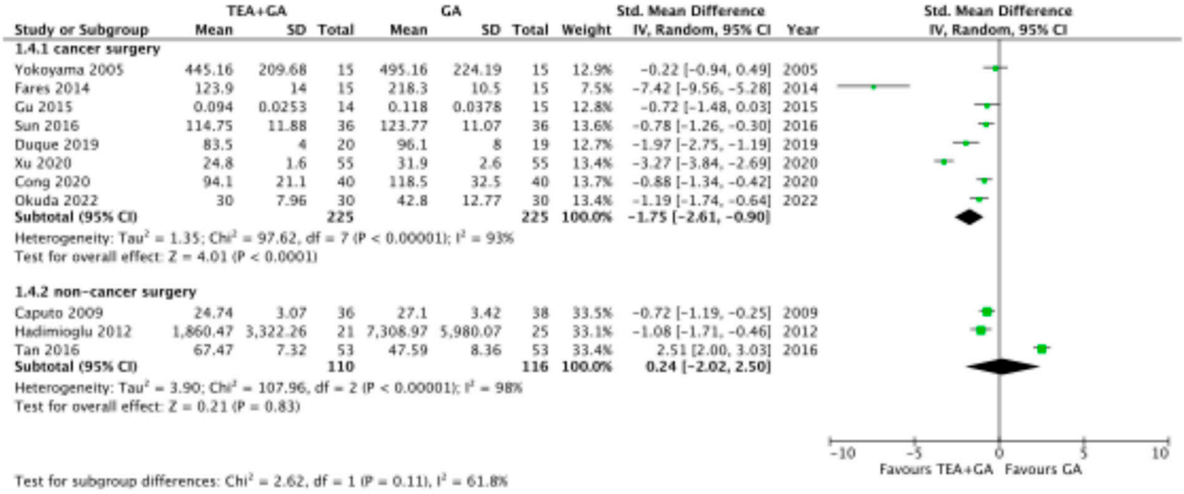

Supplementary Figure S4. Serum level of IL-6 at the end of surgery. (A) Overall analysis of IL-6 level at the end of surgery. (B) Subgroup analysis of IL-6 level at the end of surgery.

A

Study or Subgroup	TEA+GA Mean	TEA+GA SD	TEA+GA Total	GA Mean	GA SD	GA Total	Weight	Std. Mean Difference IV, Random, 95% CI	Year
Yokoyama 2005	2.68	1.69	15	2.35	1.85	15	14.2%	0.18 [−0.54, 0.90]	2005
Hadimioglu 2012	1,842.52	496.06	21	7,677.17	921.26	25	8.6%	−7.56 [−9.28, −5.85]	2012
Tan 2016	26.05	3.95	53	29.63	3.33	53	15.6%	−0.97 [−1.38, −0.57]	2016
Pi 2019	0.77	0.1	74	0.8	0.1	75	15.9%	−0.30 [−0.62, 0.02]	2019
Liu 2019	770.35	99.56	54	799.56	192.21	53	15.7%	−0.19 [−0.57, 0.19]	2019
Duque 2019	0.81	0.1	20	0.74	0.1	19	14.5%	0.69 [0.04, 1.33]	2019
Cong 2020	33.5	4.8	40	35.8	8.5	40	15.5%	−0.33 [−0.77, 0.11]	2020
**Total (95% CI)**			**277**			**280**	**100.0%**	**−0.80 [−1.53, −0.08]**	

Heterogeneity: $Tau^2 = 0.84$; $Chi^2 = 89.51$, df = 6 ($P < 0.00001$); $I^2 = 93\%$
Test for overall effect: Z = 2.17 (P = 0.03)

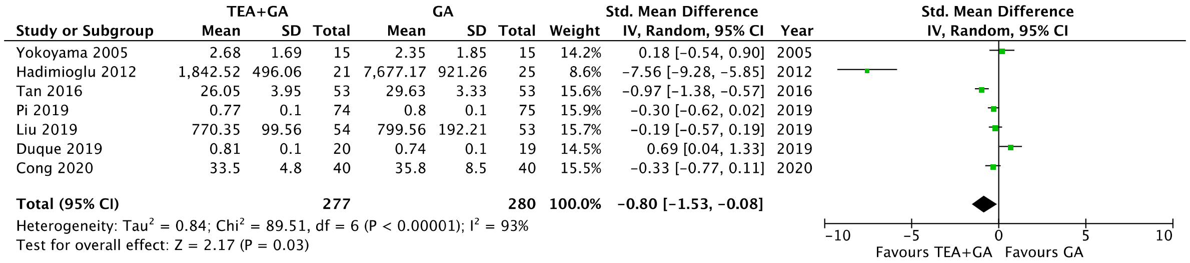

B

Study or Subgroup	TEA+GA Mean	TEA+GA SD	TEA+GA Total	GA Mean	GA SD	GA Total	Weight	Std. Mean Difference IV, Random, 95% CI	Year
**2.4.1 cancer surgery**									
Yokoyama 2005	2.68	1.69	15	2.35	1.85	15	12.5%	0.18 [−0.54, 0.90]	2005
Liu 2019	770.35	99.56	54	799.56	192.21	53	24.4%	−0.19 [−0.57, 0.19]	2019
Pi 2019	0.77	0.1	74	0.8	0.1	75	27.2%	−0.30 [−0.62, 0.02]	2019
Duque 2019	0.81	0.1	20	0.74	0.1	19	14.3%	0.69 [0.04, 1.33]	2019
Cong 2020	33.5	4.8	40	35.8	8.5	40	21.6%	−0.33 [−0.77, 0.11]	2020
**Subtotal (95% CI)**			**203**			**202**	**100.0%**	**−0.08 [−0.39, 0.23]**	
Heterogeneity: $Tau^2 = 0.06$; $Chi^2 = 8.70$, df = 4 (P = 0.07); $I^2 = 54\%$									
Test for overall effect: Z = 0.50 (P = 0.62)									
**2.4.2 non-cancer surgery**									
Hadimioglu 2012	1,842.52	496.06	21	7,677.17	921.26	25	49.2%	−7.56 [−9.28, −5.85]	2012
Tan 2016	26.05	3.95	53	29.63	3.33	53	50.8%	−0.97 [−1.38, −0.57]	2016
**Subtotal (95% CI)**			**74**			**78**	**100.0%**	**−4.21 [−10.67, 2.24]**	
Heterogeneity: $Tau^2 = 21.32$; $Chi^2 = 53.62$, df = 1 ($P < 0.00001$); $I^2 = 98\%$									
Test for overall effect: Z = 1.28 (P = 0.20)									

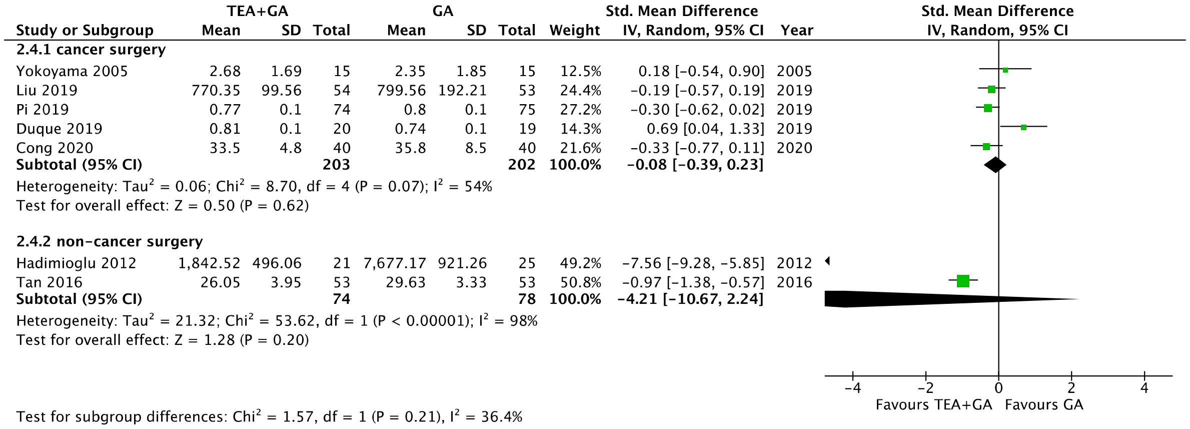

Test for subgroup differences: $Chi^2 = 1.57$, df = 1 (P = 0.21), $I^2 = 36.4\%$

Supplementary Figure S5. Serum level of TNF-α at the end of surgery. (A) Overall analysis of TNF-α level at the end of surgery. (B) Subgroup analysis of TNF-α level at the end of surgery.

A

Study or Subgroup	TEA+GA Mean	TEA+GA SD	TEA+GA Total	GA Mean	GA SD	GA Total	Weight	Std. Mean Difference IV, Random, 95% CI	Year
Yokoyama 2005	4.43	2.1	15	4.17	2.17	15	20.0%	0.12 [−0.60, 0.83]	2005
Sidiropoulou 2016	4.58	1.14	30	4.91	1.16	30	20.8%	−0.28 [−0.79, 0.23]	2016
Pi 2019	2.04	0.1	74	2.3	0.11	75	21.0%	−2.46 [−2.89, −2.03]	2019
Liu 2019	2.097	0.199	54	2.3	0.196	53	21.1%	−1.02 [−1.42, −0.62]	2019
Duque 2019	0.5	0.1	20	1.6	0.3	19	17.1%	−4.87 [−6.17, −3.57]	2019
**Total (95% CI)**			**193**			**192**	**100.0%**	**−1.60 [−2.78, −0.42]**	

Heterogeneity: $Tau^2 = 1.69$; $Chi^2 = 89.37$, df = 4 ($P < 0.00001$); $I^2 = 96\%$
Test for overall effect: Z = 2.65 (P = 0.008)

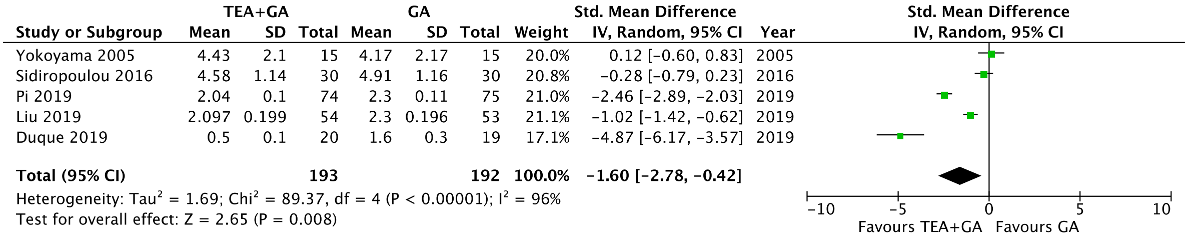

B

Study or Subgroup	TEA+GA Mean	TEA+GA SD	TEA+GA Total	GA Mean	GA SD	GA Total	Weight	Std. Mean Difference IV, Random, 95% CI	Year
**3.4.1 cancer surgery**									
Yokoyama 2005	4.43	2.1	15	4.17	2.17	15	25.3%	0.12 [−0.60, 0.83]	2005
Pi 2019	2.04	0.1	74	2.3	0.11	75	26.4%	−2.46 [−2.89, −2.03]	2019
Liu 2019	2.097	0.199	54	2.3	0.196	53	26.4%	−1.02 [−1.42, −0.62]	2019
Duque 2019	0.5	0.1	20	1.6	0.3	19	22.0%	−4.87 [−6.17, −3.57]	2019
**Subtotal (95% CI)**			**163**			**162**	**100.0%**	**−1.96 [−3.37, −0.55]**	
Heterogeneity: $Tau^2 = 1.92$; $Chi^2 = 69.77$, df = 3 ($P < 0.00001$); $I^2 = 96\%$									
Test for overall effect: Z = 2.72 (P = 0.007)									
**3.4.2 non-cancer surgery**									
Sidiropoulou 2016	4.58	1.14	30	4.91	1.16	30	100.0%	−0.28 [−0.79, 0.23]	2016
**Subtotal (95% CI)**			**30**			**30**	**100.0%**	**−0.28 [−0.79, 0.23]**	
Heterogeneity: Not applicable									
Test for overall effect: Z = 1.09 (P = 0.28)									

Test for subgroup differences: $Chi^2 = 4.79$, df = 1 ($P = 0.03$), $I^2 = 79.1\%$

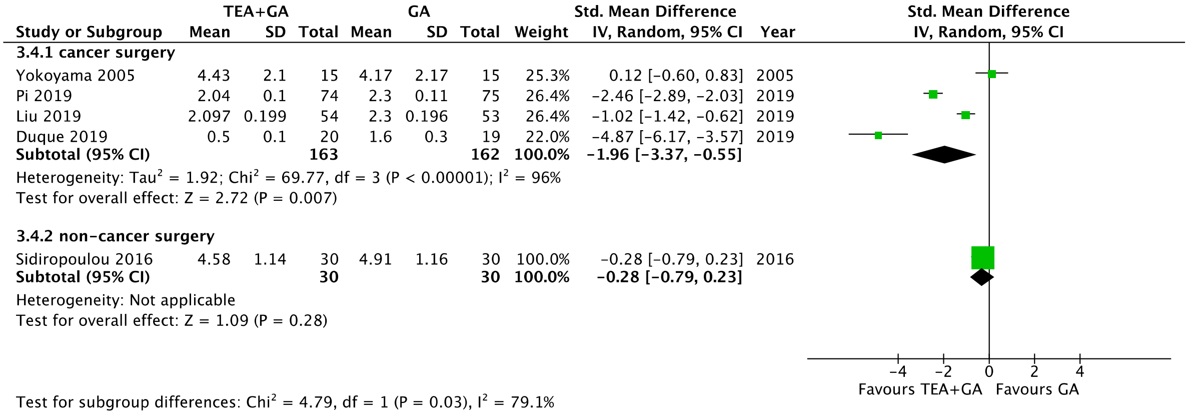

Supplementary Figure S6. Serum level of CRP at the end of surgery. (A) Overall analysis of CRP level at the end of surgery. (B) Subgroup analysis of CRP level at the end of surgery.

A

Study or Subgroup	TEA+GA Mean	TEA+GA SD	TEA+GA Total	GA Mean	GA SD	GA Total	Weight	Std. Mean Difference IV, Random, 95% CI	Year
Yokoyama 2005	10.22	3.51	15	13.28	5.15	15	10.5%	−0.68 [−1.41, 0.06]	2005
Gu 2015	216.6	73.5	14	273.8	54.8	15	10.2%	−0.86 [−1.63, −0.10]	2015
Sidiropoulou 2016	12.8	4.4	30	15.9	5.8	30	12.9%	−0.59 [−1.11, −0.08]	2016
Sun 2016	172.02	34.07	36	201.11	40.72	36	13.3%	−0.77 [−1.25, −0.29]	2016
Hou 2019	263.76	52.32	60	340.85	55.34	60	14.1%	−1.42 [−1.82, −1.02]	2019
Duque 2019	19.9	1.6	20	20.7	1.6	19	11.6%	−0.49 [−1.13, 0.15]	2019
Xu 2020	137.37	4.7	55	138.1	5.79	55	14.4%	−0.14 [−0.51, 0.24]	2020
Cong 2020	160.7	50.9	40	235.5	41.5	40	13.0%	−1.60 [−2.10, −1.09]	2020
**Total (95% CI)**			**270**			**270**	**100.0%**	**−0.82 [−1.22, −0.43]**	

Heterogeneity: $Tau^2 = 0.25$; $Chi^2 = 32.37$, df = 7 ($P < 0.0001$); $I^2 = 78\%$
Test for overall effect: Z = 4.06 ($P < 0.0001$)

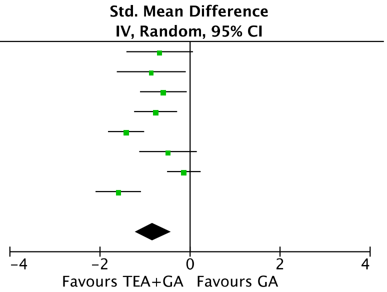

B

Study or Subgroup	TEA+GA Mean	TEA+GA SD	TEA+GA Total	GA Mean	GA SD	GA Total	Weight	Std. Mean Difference IV, Random, 95% CI	Year
Yokoyama 2005	19.33	9.7	15	24.55	11.05	15	14.3%	−0.49 [−1.22, 0.24]	2005
Gu 2015	166.9	46.8	14	210	67	15	14.3%	−0.72 [−1.48, 0.03]	2015
Sidiropoulou 2016	19.9	6.7	30	27.3	9.4	30	14.6%	−0.89 [−1.43, −0.36]	2016
Sun 2016	161.22	29.09	36	178.67	34.07	36	14.7%	−0.54 [−1.02, −0.07]	2016
Duque 2019	28.2	2.5	20	33.5	2.4	19	14.2%	−2.12 [−2.92, −1.32]	2019
Cong 2020	175.6	56.6	40	210.8	43.6	40	14.8%	−0.69 [−1.14, −0.24]	2020
Xu 2020	147.15	4.34	55	197.54	6.8	55	13.1%	−8.77 [−10.01, −7.53]	2020
**Total (95% CI)**			**210**			**210**	**100.0%**	**−1.94 [−3.19, −0.68]**	

Heterogeneity: $Tau^2 = 2.74$; $Chi^2 = 166.40$, df = 6 ($P < 0.00001$); $I^2 = 96\%$
Test for overall effect: Z = 3.02 ($P = 0.003$)

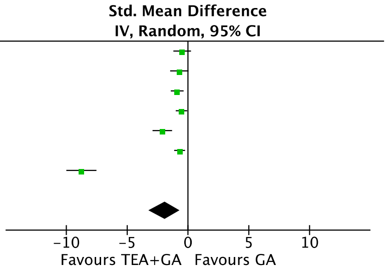

Supplementary Figure S7. Serum level of cortisol after surgery. Forest plot of cortisol at (A) 24 hours after surgery and (B) the end of surgery.

A

Study or Subgroup	TEA+GA Mean	TEA+GA SD	TEA+GA Total	GA Mean	GA SD	GA Total	Weight	Std. Mean Difference IV, Random, 95% CI	Year
Yokoyama 2005	0.66	0.37	15	0.77	0.52	15	20.5%	−0.24 [−0.96, 0.48]	2005
Moselli 2011	55.01	12.23	18	96.15	21.64	17	19.4%	−2.31 [−3.18, −1.43]	2011
Liu 2019	2,018.72	138	54	2,127.75	142	53	22.3%	−0.77 [−1.17, −0.38]	2019
Duque 2019	8.69	0.2	20	9.83	0.2	19	15.2%	−5.58 [−7.03, −4.13]	2019
Pi 2019	2.01	0.15	74	2.13	0.14	75	22.6%	−0.82 [−1.16, −0.49]	2019
**Total (95% CI)**			**181**			**179**	**100.0%**	**−1.70 [−2.66, −0.74]**	

Heterogeneity: $Tau^2 = 1.04$; $Chi^2 = 53.51$, df = 4 ($P < 0.00001$); $I^2 = 93\%$
Test for overall effect: Z = 3.47 (P = 0.0005)

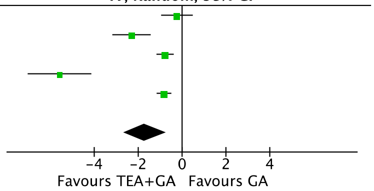

B

Study or Subgroup	TEA + GA Mean	TEA + GA SD	TEA + GA Total	GA Mean	GA SD	GA Total	Weight	Std. Mean Difference IV, Random, 95% CI
Cong 2020	5.6	2.4	40	7.8	3.4	40	20.6%	−0.74 [−1.19, −0.29]
Duque 2019	0.83	0.1	20	0.46	0.1	19	19.8%	3.62 [2.57, 4.68]
Gu 2015	6.3	3	14	6.9	2	15	20.3%	−0.23 [−0.96, 0.50]
Moselli 2011	90.46	14.67	18	33.65	9.62	17	19.3%	4.45 [3.16, 5.74]
Wang 2019	18.19	1.2	20	24.59	3.03	20	20.1%	−2.72 [−3.60, −1.84]
**Total (95% CI)**			**112**			**111**	**100.0%**	**0.83 [−1.26, 2.91]**

Heterogeneity: $Tau^2 = 5.44$; $Chi^2 = 137.14$, df = 4 ($P < 0.00001$); $I^2 = 97\%$
Test for overall effect: Z = 0.78 (P = 0.44)

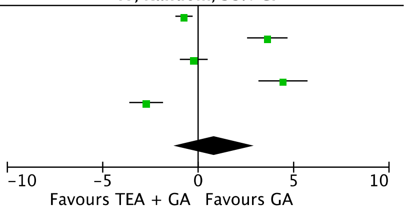

C

Study or Subgroup	TEA+GA Mean	TEA+GA SD	TEA+GA Total	GA Mean	GA SD	GA Total	Weight	Std. Mean Difference IV, Random, 95% CI	Year
Caputo 2009	14.74	1.05	36	16.2	0.98	38	17.8%	−1.42 [−1.94, −0.91]	2009
Fares 2014	11.7	1.4	15	18.5	4.9	15	13.2%	−1.84 [−2.71, −0.96]	2014
Duque 2019	2.09	0.4	20	5.1	3	19	15.2%	−1.40 [−2.10, −0.69]	2019
Liu 2019	3.2	0.199	54	3.504	0.292	53	19.0%	−1.21 [−1.62, −0.80]	2019
Pi 2019	3.2	0.19	74	3.8	0.29	75	18.9%	−2.43 [−2.86, −2.01]	2019
Elsayed 2020	15.9	1.6	20	17.3	1.59	20	16.0%	−0.86 [−1.51, −0.21]	2020
**Total (95% CI)**			**219**			**220**	**100.0%**	**−1.53 [−2.04, −1.03]**	

Heterogeneity: $Tau^2 = 0.30$; $Chi^2 = 24.07$, df = 5 ($P = 0.0002$); $I^2 = 79\%$
Test for overall effect: Z = 5.98 (P < 0.00001)

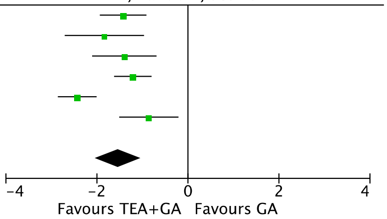

D

Study or Subgroup	TEA+GA Mean	TEA+GA SD	TEA+GA Total	GA Mean	GA SD	GA Total	Weight	Std. Mean Difference IV, Random, 95% CI	Year
Yokoyama 2005	28.47	15.27	15	24.97	17.65	15	21.1%	0.21 [−0.51, 0.92]	2005
Moselli 2011	476.77	25.67	18	213.94	28.85	17	14.5%	9.42 [6.99, 11.86]	2011
Xu 2014	24.3	6.5	20	17.2	5.43	20	21.2%	1.16 [0.49, 1.84]	2014
Sun 2016	8.15	0.91	36	10.08	1.34	36	21.5%	−1.67 [−2.21, −1.13]	2016
Hou 2019	10.54	2.38	60	12.68	1.46	60	21.8%	−1.08 [−1.46, −0.69]	2019
**Total (95% CI)**			**149**			**148**	**100.0%**	**1.06 [−0.52, 2.64]**	

Heterogeneity: $Tau^2 = 2.96$; $Chi^2 = 116.98$, df = 4 ($P < 0.00001$); $I^2 = 97\%$
Test for overall effect: Z = 1.31 (P = 0.19)

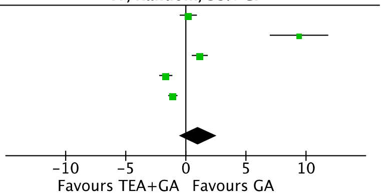

Supplementary Figure S8. Forest plot of IL-1β(A), IL-4 (B), IL-8(C), and IL-10 (D) level at 24 hours after surgery.

Search Strategy:
((“TNF”[MeSH Terms]) OR (“TNF”)) OR ((“Interleukin”[MeSH Terms]) OR (“Interkeukin”)) OR ((“cytokine”[MeSH Terms]) OR (“cytokine”)) OR ((“glucocorticoid”[MeSH Terms]) OR (“glucocorticoid”) OR (“cortisol”)) OR ((“C-reactive protein ”[MeSH Terms]) OR (“C-reactive protein”))
AND
((“Analgesia, Epidural”[MeSH Terms]) OR (“Anesthesia, Epidural”[MeSH Terms]) OR (“Epidural”))

Risk Assessment

Caputo 2008- low risk of bias for all domains

1- Randomization process: LOW RISK
1.1 allocation sequence random- Y
1.2 concealed- Y
1.3 baseline differences-probably no (PN)

2. Deviation from intended interventions: LOW RISK
2.1 participants aware of their assigned intervention – probably yes (PY)
2.2 carers aware of assigned intervention - Y
2.3 deviations arising from trial context - PN

3. Missing outcome data: LOW RISK
3.1 data available for nearly all participants- PY

4.Measurement of outcome: LOW RISK
4.1 method of measuring outcome inappropriate - N
4.2 could measurement of outcome differed between intervention groups - N
4.3 were outcome assessors aware of intervention? PY, outcome assessors might not be blind
4.4 could assessment of outcome been influenced by knowledge of intervention group- PN (objective endpoints)

5. Selection of reported result: LOW RISK
5.1 was the data that produced this result analyzed in accordance with a pre-specified analysis plan that was finalized before unblinded outcome data were available for analysis - PY
5.2 were only certain outcome measurements reported- PN
5.3 multiple analysis of data- PN

Cong 2020 - some concerns for ONE domain

1- Randomization process: SOME CONCERNS
1.1 allocation sequence random - Y
1.2 concealed - NI
1.3 baseline differences - N

2. Deviation from intended interventions: LOW RISK

2.1 participants aware of their assigned intervention - PY
2.2 carers aware of assigned intervention - Y
2.3 deviations arising from trial context - PN

3. Missing outcome data: LOW RISK
3.1 data available for nearly all participants - PY

4.Measurement of outcome: LOW RISK
4.1 method of measuring outcome inappropriate - N
4.2 could measurement of outcome differed between intervention groups - N
4.3 were outcome assessors aware of intervention - PY, outcome assessors might not be blind
4.4 could assessment of outcome been influenced by knowledge of intervention group- PN (objective endpoints)

5. Selection of reported result: LOW RISK
5.1 was the data that produced this result analyzed in accordance with a pre-specified analysis plan that was finalized before unblinded outcome data were available for analysis - PY
5.2 were only certain outcome measurements reported- PN
5.3 multiple analysis of data- PN

Duque 2019 – high risk for one domain and some concerns for one domain
1. Randomization process: HIGH RISK
1.1 allocation sequence random- Y
1.2 concealed from PI- PN, expertise-based randomization was done
1.3 baseline differences - PN

2. Deviation from intended interventions: SOME CONCERNS
2.1 participants aware of their assigned intervention - PY
2.2 carers aware of assigned intervention - Y
2.3 deviations arising from trial context – Y, few patients converted to other group
2.4 deviations likely to have affected outcome- PN

3. Missing outcome data: LOW RISK
3.1 data available for nearly all participants- PY

4.Measurement of outcome: LOW RISK
4.1 method of measuring outcome inappropriate - N
4.2 could measurement of outcome differed between intervention groups - N
4.3 were outcome assessors aware of intervention- N
4.4 could assessment of outcome been influenced by knowledge of intervention group- PN (objective endpoints)

5. Selection of reported result: LOW RISK

5.1 was the data that produced this result analyzed in accordance with a pre-specified analysis plan that was finalized before unblinded outcome data were available for analysis - PY
5.2 were only certain outcome measurements reported- PN
5.3 multiple analysis of data- PN

Elsayed- some concerns in one domain
1. Randomization process: SOME CONCERNS
1.1 allocation sequence random- PY
1.2 concealed - NI
1.3 baseline differences - PN

2. Deviation from intended interventions: LOW RISK
2.1 participants aware of their assigned intervention - PY
2.2 carers aware of assigned intervention - Y
2.3 deviations arising from trial context - PN

3. Missing outcome data: LOW RISK
3.1 data available for nearly all participants- PY

4. Measurement of outcome: LOW RISK
4.1 method of measuring outcome inappropriate - N
4.2 could measurement of outcome differed between intervention groups - N
4.3 were outcome assessors aware of intervention- N
4.4 could assessment of outcome been influenced by knowledge of intervention group- PN (objective endpoints)

5. Selection of reported result: LOW RISK
5.1 was the data that produced this result analyzed in accordance with a pre-specified analysis plan that was finalized before unblinded outcome data were available for analysis - PY
5.2 were only certain outcome measurements reported- PN
5.3 multiple analysis of data- PN

Fant 2013- low risk
1. Randomization process: LOW RISK
1.1 allocation sequence random- Y
1.2 concealed - Y
1.3 baseline differences - N

2. Deviation from intended interventions: LOW RISK
2.1 participants aware of their assigned intervention - Y
2.2 carers aware of assigned intervention - Y
2.3 deviations arising from trial context - PN

3. Missing outcome data: LOW RISK

3.1 data available for nearly all participants- Y

4. Measurement of outcome: LOW RISK
4.1 method of measuring outcome inappropriate - N
4.2 could measurement of outcome differed between intervention groups - N
4.3 were outcome assessors aware of intervention- N
4.4 could assessment of outcome been influenced by knowledge of intervention group- PN (objective endpoints)

5. Selection of reported result: LOW RISK
5.1 was the data that produced this result analyzed in accordance with a pre-specified analysis plan that was finalized before unblinded outcome data were available for analysis - PY
5.2 were only certain outcome measurements reported- PN
5.3 multiple analysis of data- PN

Fares 2014-some concerns for one domain
1. Randomization process: LOW RISK
1.1 allocation sequence random - Y
1.2 concealed - Y
1.3 baseline differences - PN

2. Deviation from intended interventions: LOW RISK
2.1 participants aware of their assigned intervention - PY
2.2 carers aware of assigned intervention - PY
2.3 deviations arising from trial context - PN

3. Missing outcome data: LOW RISK
3.1 data available for nearly all participants - PY

4.Measurement of outcome: LOW RISK
4.1 method of measuring outcome inappropriate - PN
4.2 could measurement of outcome differed between intervention groups - PN
4.3 were outcome assessors aware of intervention- PN
4.4 could assessment of outcome been influenced by knowledge of intervention group- N (objective endpoint)

5. Selection of reported result: LOW RISK
5.1 was the data that produced this result analyzed in accordance with a pre-specified analysis plan that was finalized before unblinded outcome data were available for analysis - PY
5.2 were only certain outcome measurements reported- PN
5.3 multiple analysis of data- PN

Gu 2015- some concerns in one domain

1. Randomization process: SOME CONCERNS
1.1 allocation sequence random - Y
1.2 concealed - NI
1.3 baseline differences - PN

2. Deviation from intended interventions: LOW RISK
2.1 participants aware of their assigned intervention - PN
2.2 carers aware of assigned intervention - PY
2.3 deviations arising from trial context - PN

3. Missing outcome data: LOW RISK
3.1 data available for nearly all participants- PY

4.Measurement of outcome: LOW RISK
4.1 method of measuring outcome inappropriate - PN
4.2 could measurement of outcome differed between intervention groups - PN
4.3 were outcome assessors aware of intervention – PN
4.4 could assessment of outcome been influenced by knowledge of intervention group- PN (objective endpoints)

5. Selection of reported result: LOW RISK
5.1 was the data that produced this result analyzed in accordance with a pre-specified analysis plan that was finalized before unblinded outcome data were available for analysis - PY
5.2 were only certain outcome measurements reported- PN
5.3 multiple analysis of data- PN

Hadimiuoglu 2012-some concerns
1. Randomization process: SOME CONCERNS
1.1 allocation sequence random- NI
1.2 concealed - NI
1.3 baseline differences - PN

2. Deviation from intended interventions: LOW RISK
2.1 participants aware of their assigned intervention - PN
2.2 carers aware of assigned intervention - PY
2.3 deviations arising from trial context - PN

3. Missing outcome data: LOW RISK
3.1 data available for nearly all participants - Y

4.Measurement of outcome: LOW RISK
4.1 method of measuring outcome inappropriate - PN
4.2 could measurement of outcome differed between intervention groups - PN
4.3 were outcome assessors aware of intervention – PN

4.4 could assessment of outcome been influenced by knowledge of intervention group- PN (objective endpoints)

5. Selection of reported result: LOW RISK
5.1 was the data that produced this result analyzed in accordance with a pre-specified analysis plan that was finalized before unblinded outcome data were available for analysis - PY
5.2 were only certain outcome measurements reported - PN
5.3 multiple analysis of data - PN

Hou 2019- some concerns
1. Randomization process: SOME CONCERNS
1.1 allocation sequence random- NI
1.2 concealed - NI
1.3 baseline differences - PN

2. Deviation from intended interventions: LOW RISK
2.1 participants aware of their assigned intervention - Y
2.2 carers aware of assigned intervention - Y
2.3 deviations arising from trial context - PN

3. Missing outcome data: LOW RISK
3.1 data available for nearly all participants - PY

4. Measurement of outcome: LOW RISK
4.1 method of measuring outcome inappropriate - PN
4.2 could measurement of outcome differed between intervention groups - PN
4.3 were outcome assessors aware of intervention – PN
4.4 could assessment of outcome been influenced by knowledge of intervention group- PN (objective endpoints)

5. Selection of reported result: LOW RISK
5.1 was the data that produced this result analyzed in accordance with a pre-specified analysis plan that was finalized before unblinded outcome data were available for analysis - PY
5.2 were only certain outcome measurements reported - PN
5.3 multiple analysis of data - PN

Liu 2019- some concerns
1. Randomization process: SOME CONCERNS
1.1 allocation sequence random- PY
1.2 concealed - NI
1.3 baseline differences - PN

2. Deviation from intended interventions: LOW RISK
2.1 participants aware of their assigned intervention - Y

2.2 carers aware of assigned intervention - Y
2.3 deviations arising from trial context - PN

3. Missing outcome data: LOW RISK
3.1 data available for nearly all participants - PY

4. Measurement of outcome: LOW RISK
4.1 method of measuring outcome inappropriate - PN
4.2 could measurement of outcome differed between intervention groups - PN
4.3 were outcome assessors aware of intervention – PN
4.4 could assessment of outcome been influenced by knowledge of intervention group- PN (objective endpoints)

5. Selection of reported result: LOW RISK
5.1 was the data that produced this result analyzed in accordance with a pre-specified analysis plan that was finalized before unblinded outcome data were available for analysis - PY
5.2 were only certain outcome measurements reported - PN
5.3 multiple analysis of data - PN

Moselli 2011- some concerns
1. Randomization process: some concerns
1.1 allocation sequence random- Y
1.2 concealed - NI
1.3 baseline differences - PN

2. Deviation from intended interventions: LOW RISK
2.1 participants aware of their assigned intervention - Y
2.2 carers aware of assigned intervention - PY
2.3 deviations arising from trial context - N

3. Missing outcome data: LOW RISK
3.1 data available for nearly all participants - PY

4. measurement of outcome: LOW RISK
4.1 method of measuring outcome inappropriate - PN
4.2 could measurement of outcome differed between intervention groups - PN
4.3 were outcome assessors aware of intervention – PN
4.4 could assessment of outcome been influenced by knowledge of intervention group- PN (objective endpoints)

5. Selection of reported result: LOW RISK
5.1 was the data that produced this result analyzed in accordance with a pre-specified analysis plan that was finalized before unblinded outcome data were available for analysis - PY
5.2 were only certain outcome measurements reported- PN

5.3 multiple analysis of data- PN

Pi2019- some concerns
1. Randomization process: SOME CONCERNS
1.1 allocation sequence random- Y
1.2 concealed - NI
1.3 baseline differences-PN

2. Deviation from intended interventions: LOW RISK
2.1 participants aware of their assigned intervention - Y
2.2 carers aware of assigned intervention - PY
2.3 deviations arising from trial context - PN

3. Missing outcome data: LOW RISK
3.1 data available for nearly all participants - PY

4. Measurement of outcome: some concerns
4.1 method of measuring outcome inappropriate - PN
4.2 could measurement of outcome differed between intervention groups - PN
4.3 were outcome assessors aware of intervention- PY
4.4 could assessment of outcome been influenced by knowledge of intervention group- PN (objective endpoints)

5. Selection of reported result: LOW RISK
5.1 was the data that produced this result analyzed in accordance with a pre-specified analysis plan that was finalized before unblinded outcome data were available for analysis - PY
5.2 were only certain outcome measurements reported - PN
5.3 multiple analysis of data - PN

Sidiropoulu 2016
1. Randomization process: SOME CONCERNS
1.1 allocation sequence random- Y
1.2 concealed - NI
1.3 baseline differences - PN

2. Deviation from intended interventions: LOW RISK
2.1 participants aware of their assigned intervention - N
2.2 carers aware of assigned intervention - PY
2.3 deviations arising from trial context - N

3. Missing outcome data: LOW RISK
3.1 data available for nearly all participants - PY

4. Measurement of outcome: some concerns

4.1 method of measuring outcome inappropriate - PN
4.2 could measurement of outcome differed between intervention groups - PN
4.3 were outcome assessors aware of intervention- PN
4.4 could assessment of outcome been influenced by knowledge of intervention group- PN (objective endpoints)

5. Selection of reported result: LOW RISK
5.1 was the data that produced this result analyzed in accordance with a pre-specified analysis plan that was finalized before unblinded outcome data were available for analysis - PY
5.2 were only certain outcome measurements reported - PN
5.3 multiple analysis of data - PN

Sun2016- some concern
1. Randomization process: SOME CONCERNS
1.1 allocation sequence random- NI
1.2 concealed - NI
1.3 baseline differences - PN

2. Deviation from intended interventions: LOW RISK
2.1 participants aware of their assigned intervention - Y
2.2 carers aware of assigned intervention - PY
2.3 deviations arising from trial context - N

3. Missing outcome data: LOW RISK
3.1 data available for nearly all participants - PY

4. Measurement of outcome: LOW RISK
4.1 method of measuring outcome inappropriate - PN
4.2 could measurement of outcome differed between intervention groups - PN
4.3 were outcome assessors aware of intervention- PN
4.4 could assessment of outcome been influenced by knowledge of intervention group- PN (objective endpoints)

5. Selection of reported result: LOW RISK
5.1 was the data that produced this result analyzed in accordance with a pre-specified analysis plan that was finalized before unblinded outcome data were available for analysis - PY
5.2 were only certain outcome measurements reported- PN
5.3 multiple analysis of data - PN

Tan 2016- LOW RISK
1. Randomization process: LOW RISK
1.1 allocation sequence random - Y

1.2 concealed - Y
1.3 baseline differences - PN

2. Deviation from intended interventions: LOW RISK
2.1 participants aware of their assigned intervention: Y
2.2 carers aware of assigned intervention - N
2.3 deviations arising from trial context - PN

3. Missing outcome data: LOW RISK
3.1 data available for nearly all participants - PY

4. Measurement of outcome: LOW RISK
4.1 method of measuring outcome inappropriate - PN
4.2 could measurement of outcome differed between intervention groups - PN
4.3 were outcome assessors aware of intervention- N
4.4 could assessment of outcome been influenced by knowledge of intervention group- PN (objective endpoints)

5. Selection of reported result: LOW RISK
5.1 was data analysed according to plan - PY
5.2 were only certain outcome measurements reported - PN
5.3 multiple analysis of data - PN

Wang 2019-some concerns
1. Randomization process: SOME CONCERNS
1.1 allocation sequence random- Y
1.2 concealed - NI
1.3 baseline differences - PN

2. Deviation from intended interventions: LOW RISK
2.1 participants aware of their assigned intervention - PY
2.2 carers aware of assigned intervention - PY
2.3 deviations arising from trial context - PN

3. Missing outcome data: LOW RISK
3.1 data available for nearly all participants - PY

4.measurement of outcome: LOW RISK
4.1 method of measuring outcome inappropriate - PN
4.2 could measurement of outcome differed between intervention groups - PN
4.3 were outcome assessors aware of intervention- N
4.4 could assessment of outcome been influenced by knowledge of intervention group- PN (objective endpoints)

5. Selection of reported result: LOW RISK
5.1 was the data that produced this result analyzed in accordance with a pre-specified analysis plan that was finalized before unblinded outcome data were available for analysis - PY
5.2 were only certain outcome measurements reported - PN
5.3 multiple analysis of data - PN

Xu2014-some concerns
1. Randomization process: SOME CONCERNS
1.1 allocation sequence random - Y
1.2 concealed - NI
1.3 baseline differences - PN

2. Deviation from intended interventions: LOW RISK
2.1 participants aware of their assigned intervention - Y
2.2 carers aware of assigned intervention - Y
2.3 deviations arising from trial context - PN

3. Missing outcome data: LOW RISK
3.1 data available for nearly all participants - PY

4. Measurement of outcome: LOW RISK
4.1 method of measuring outcome inappropriate - PN
4.2 could measurement of outcome differed between intervention groups - PN
4.3 were outcome assessors aware of intervention – PN
4.4 could assessment of outcome been influenced by knowledge of intervention group- PN (objective endpoints)

5. Selection of reported result: LOW RISK
5.1 was the data that produced this result analyzed in accordance with a pre-specified analysis plan that was finalized before unblinded outcome data were available for analysis - PY
5.2 were only certain outcome measurements reported- PN
5.3 multiple analysis of data - PN

Xu2020-low risk
1. Randomization process: LOW RISK
1.1 allocation sequence random- Y
1.2 concealed - Y
1.3 baseline differences - PN

2. Deviation from intended interventions: LOW RISK
2.1 participants aware of their assigned intervention - Y
2.2 carers aware of assigned intervention - Y
2.3 deviations arising from trial context - PN

3. Missing outcome data: LOW RISK
3.1 data available for nearly all participants - PY

4. Measurement of outcome: LOW RISK
4.1 method of measuring outcome inappropriate - PN
4.2 could measurement of outcome differed between intervention groups - PN
4.3 were outcome assessors aware of intervention – PN
4.4 could assessment of outcome been influenced by knowledge of intervention group- PN (objective endpoints)

5. Selection of reported result: LOW RISK
5.1 was the data that produced this result analyzed in accordance with a pre-specified analysis plan that was finalized before unblinded outcome data were available for analysis - PY
5.2 were only certain outcome measurements reported- PN
5.3 multiple analysis of data- PN

Yan 2019 -some concerns
1. Randomization process: SOME CONCERNS
1.1 allocation sequence random - Y
1.2 concealed - NI
1.3 baseline differences - PN

2. Deviation from intended interventions: LOW RISK
2.1 participants aware of their assigned intervention - Y
2.2 carers aware of assigned intervention - Y
2.3 deviations arising from trial context - PN

3. Missing outcome data: LOW RISK
3.1 data available for nearly all participants- PY

4. Measurement of outcome: LOW RISK
4.1 method of measuring outcome inappropriate - PN
4.2 could measurement of outcome differed between intervention groups - PN
4.3 were outcome assessors aware of intervention – PN
4.4 could assessment of outcome been influenced by knowledge of intervention group- PN (objective endpoints)

5. Selection of reported result: LOW RISK
5.1 was the data that produced this result analyzed in accordance with a pre-specified analysis plan that was finalized before unblinded outcome data were available for analysis - PY
5.2 were only certain outcome measurements reported - PN
5.3 multiple analysis of data - PN

Yokoyama 2005- some concerns

1. Randomization process: SOME CONCERNS
1.1 allocation sequence random - Y
1.2 concealed - NI
1.3 baseline differences - PN

2. Deviation from intended interventions: LOW RISK
2.1 participants aware of their assigned intervention - Y
2.2 carers aware of assigned intervention - Y
2.3 deviations arising from trial context - PN

3. Missing outcome data: LOW RISK
3.1 data available for nearly all participants- PY

4. Measurement of outcome: LOW RISK
4.1 method of measuring outcome inappropriate - PN
4.2 could measurement of outcome differed between intervention groups - PN
4.3 were outcome assessors aware of intervention- N
4.4 could assessment of outcome been influenced by knowledge of intervention group- PN

5. Selection of reported result: LOW RISK
5.1 was the data that produced this result analyzed in accordance with a pre-specified analysis plan that was finalized before unblinded outcome data were available for analysis - PY
5.2 were only certain outcome measurements reported - PN
5.3 multiple analysis of data - PN

Excluded Studies

pubmed ID	Year	Author	Reason
28921063	1997	Yorozu(*1*)	no selected primary outcome reported
17218378	2007	Kawasaki(*2*)	TNF-alpha not reported as serum level
22791944	2012	Zhou(*3*)	no selected primary outcome reported
24909481	2013	Kahveci(*4*)	No information about epidural procedure
23514874	2013	Ezhevskaya(*5*)	Cytokine levels are expressed as median + range
23353589	2013	Chloropoulou(*6*)	Cytokine levels are expressed as median + range
27251956	2015	Ozcan(*7*)	Study designed for comparing different local anesthetics used in TEA

25579161	2015	Chen(*8*)	Reported time point did not meet the inclusion criteria
28078860	2016	Xu(*9*)	No information about epidural procedure
28935762	2017	Han (*10*)	Retrospective study
28868167	2017	Karadeniz(*11*)	Cytokine levels are expressed as median + range
27829181	2017	Tyagi(*12*)	Cytokine levels are expressed as median + range
28272202	2017	Zhu(*13*)	no selected primary outcome reported
29363103	2018	Li(*14*)	no selected primary outcome reported

References

1. T. Yorozu *et al.*, Epidural anesthesia during hysterectomy diminishes postoperative pain and urinary cortisol release. *Journal of anesthesia* **11**, 260-264 (1997).
2. T. Kawasaki, M. Ogata, C. Kawasaki, K. Okamoto, T. Sata, Effects of epidural anaesthesia on surgical stress-induced immunosuppression during upper abdominal surgery. *British journal of anaesthesia* **98**, 196-203 (2007).
3. D. Zhou *et al.*, Effects of anesthetic methods on preserving anti-tumor T-helper polarization following hepatectomy. *World journal of gastroenterology* **18**, 3089-3098 (2012).
4. K. Kahveci *et al.*, The effect of anesthesia type on stress hormone response: comparison of general versus epidural anesthesia. *Nigerian journal of clinical practice* **17**, 523-527 (2014).
5. A. A. Ezhevskaya, S. G. Mlyavykh, D. G. Anderson, Effects of continuous epidural anesthesia and postoperative epidural analgesia on pain management and stress response in patients undergoing major spinal surgery. *Spine* **38**, 1324-1330 (2013).
6. P. Chloropoulou *et al.*, Epidural anesthesia followed by epidural analgesia produces less inflammatory response than spinal anesthesia followed by intravenous morphine analgesia in patients with total knee arthroplasty. *Medical science monitor : international medical journal of experimental and clinical research* **19**, 73-80 (2013).
7. S. Ozcan, A. B. Ozer, M. A. Yasar, O. L. Erhan, Effects of combined general anesthesia and thoracic epidural analgesia on cytokine response in patients undergoing laparoscopic cholecystectomy. *Nigerian journal of clinical practice* **19**, 436-442 (2016).
8. W. K. Chen *et al.*, General anesthesia combined with epidural anesthesia ameliorates the effect of fast-track surgery by mitigating immunosuppression and facilitating intestinal functional recovery in colon cancer patients. *International journal of colorectal disease* **30**, 475-481 (2015).
9. Y. Xu, Y. Sun, H. Chen, Y. Wang, G. N. Wang, Effects of two different anesthetic methods on cellular immunity of patients after liver cancer resection. *Journal of biological regulators and homeostatic agents* **30**, 1099-1106 (2016).
10. X. R. Han *et al.*, Effect of different anesthetic methods on cellular immune functioning and the prognosis of patients with ovarian cancer undergoing oophorectomy. *Biosci Rep* **37**, (2017).

11. M. S. Karadeniz, O. Mammadov, H. Çiftci, S. A. Usta, K. Pembeci, Comparing the Effects of Combined General/Epidural Anaesthesia and General Anaesthesia on Serum Cytokine Levels in Radical Cystectomy. *Turkish journal of anaesthesiology and reanimation* **45**, 203-209 (2017).
12. A. Tyagi, A. Bansal, S. Das, A. K. Sethi, A. Kakkar, Effect of thoracic epidural block on infection-induced inflammatory response: A randomized controlled trial. *Journal of critical care* **38**, 6-12 (2017).
13. J. Zhu, X. R. Zhang, H. Yang, Effects of combined epidural and general anesthesia on intraoperative hemodynamic responses, postoperative cellular immunity, and prognosis in patients with gallbladder cancer: A randomized controlled trial. *Medicine* **96**, e6137 (2017).
14. M. H. Li *et al.*, Effect of combined epidural anaesthesia on tumor-infiltrating lymphocytes in lung adenocarcinoma: a prospective exploratory sub-analysis. *Acta anaesthesiologica Scandinavica* **62**, 687-700 (2018).
